# MORE OR LESS? A DECADE’S TREND OF SOCIAL PARTICIPATION AMONG CHINESE OLDER ADULTS: BASED ON A LONGITUDINAL SURVEY

**DOI:** 10.1093/geroni/igz038.2348

**Published:** 2019-11-08

**Authors:** Chenxin Tan, Yun Zhou

**Affiliations:** Department of Sociology, Peking University, Beijing, China

## Abstract

Social participation is of great significance in healthy aging. While studies on social participation among Chinese elderly are growing, there is a lack of understanding the changes over time of the participation. Using datasets from the Chinese Longitudinal Healthy Longevity Survey (CLHLS), this paper presents a comprehensive analysis on a decade’s trend of social participation among Chinese older adults. First, we use the method of Latent Class Analysis (LCA) to identify types of social participation; in this study, we concluded three types, no participation, the family-centered, and the society-oriented. Second, we examine the characteristics of the elderly by types of participation in terms of demographic, socioeconomic and health condition and analyze the changes in the characteristics over time. And third, we interpret the trend of social participation with broader social environment, or the fluctuant structural and institutional differences under the context of China’s unique social system. Our general conclusion is that while the overall level of participation holds relatively steady, there is a dynamic micro progress and complex mechanisms in this long period. In addition, although both the family-centered participants and the society-oriented possess broader scopes of social participation, the related attributes are different across time. This paper contributes to our knowledge of life of the elderly under the circumstances of fast aging process in China.

